# Resident and Attending Physicians’ Perceptions of Patient Access to Provider Notes: Comparison of Perceptions Prior to Pilot Implementation

**DOI:** 10.2196/mededu.8904

**Published:** 2018-06-15

**Authors:** Deepa Rani Nandiwada, Gary S Fischer, Glenn Updike, Margaret B Conroy

**Affiliations:** ^1^ Department of Medicine University of Pennsylvania Perelman School of Medicine Philadelphia, PA United States; ^2^ Division of General Internal Medicine University of Pittsburgh School of Medicine Pittsburgh, PA United States; ^3^ Department of Obstetrics, Gynecology, & Reproductive Sciences University of Pittsburgh Pittsburgh, PA United States; ^4^ Department of Medicine University of Utah Salt Lake City, UT United States

**Keywords:** access to information, electronic health records, physicians, internal medicine, surveys and questionnaires

## Abstract

**Background:**

As electronic health records have become a more integral part of a physician’s daily life, new electronic health record tools will continue to be rolled out to trainees. Patient access to provider notes is becoming a more widespread practice because this has been shown to increase patient empowerment.

**Objective:**

In this analysis, we compared differences between resident and attending physicians’ perceptions prior to implementation of patient access to provider notes to facilitate optimal use of electronic health record features and as a potential for patient empowerment.

**Methods:**

This was a single-site study within an academic internal medicine program. Prior to implementation of patient access to provider notes, we surveyed resident and attending physicians to assess differences in perceptions of this new electronic health record tool using an open access survey provided by OpenNotes.

**Results:**

We surveyed 37% (20/54 total) of resident physicians and obtained a 100% response rate and 72% (31/44 total) of attending physicians. Similarities between the groups included concerns about documenting sensitive topics and anticipation of improved patient engagement. Compared with attending physicians, resident physicians were more concerned about litigation, discussing weight, offending patients, and communicated less overall with patients through electronic health record.

**Conclusions:**

Patient access to provider notes has the potential to empower patients but concerns of the resident physicians need to be validated and addressed prior to its utilization.

## Introduction

Electronic health records (EHRs) have become a part of daily life for physicians practicing in today’s technological era. EHRs are used for documentation and billing but can also increase patient engagement through portals that allow patients to contact physicians, review lab work, and perform other tasks. Recent studies have shown that patients with access to their notes feel more engaged to work as a team with their health care providers. In 2012, the OpenNotes study gave nearly 20,000 patients access to their clinical notes through a patient portal. Overall, the study showed that patients were empowered by access to their notes and were more likely to follow their respective care plans. Provider concerns regarding increased time burden, patient concerns about note content, and documentation challenges were less significant than anticipated. In fact, most providers opted to continue offering their patients access to their notes after the study period concluded [1,2,3,4].

The initial OpenNotes evaluations did not evaluate perceptions of the resident physicians. As use of open clinical notes becomes more prevalent in both community and academic centers, it is imperative to evaluate perceptions of providers at all levels of training to identify barriers for comfort using OpenNotes and opportunities for education. Few studies till date have assessed internal medicine residents’ perceptions of open clinical notes and compared their perceptions with those of attending physicians [5].

In this study, we evaluated differences in the perceptions of resident and attending physicians prior to implementation of patient access to provider notes to identify potential targets for curricular interventions and facilitate optimal use of EHR features while increasing patient empowerment.

## Methods

### Study Design

Since November 2014, office notes in our primary academic general internal medicine clinic were made available to patients through our secure patient portal. The faculty practice clinic had 54 resident and 44 attending physicians when the pilot began. Attending physicians had 3 faculty meetings set up in the months prior to roll-out of the pilot to provide feedback and address concerns related to implementation of OpenNotes. A tip sheet derived from the OpenNotes Frequently Asked Questions resources was provided and reviewed prior to roll-out. Residents had a 20-min introduction session immediately before pilot roll-out and were also provided a tip sheet. The session and tip sheets for both groups of doctors informed them how they could document sensitive topics in a special section of the chart that would remain inaccessible to patients. A standardized survey which is publically available was provided to all physicians to assess their perceptions of current practices, benefits, patient impact, and barriers to the use of open clinical notes prior to roll-out. There were 3 free response comment sections within the survey. The OpenNotes provider survey covered many possible perceived barriers such as time, addressing sensitive issues in notes, and liability. Live surveys were distributed to the attending physicians during their regularly scheduled faculty meetings and were given to the resident physicians at the start of the 20-min introduction session. This study was reviewed by the University of Pittsburgh Quality Improvement (QI) Review Board and was deemed a QI project; therefore, it was exempt from review by the Institutional Review Board. Participation in the survey was optional.

### Statistical Analysis

Descriptive statistics were generated evaluating the frequency of each response. Fisher’s exact and Chi-square tests were used to determine significant differences between the responses of the attending and resident physicians. We collapsed categories of responses on survey items so that “Disagree” included disagree and somewhat disagree and “Agree” included agree and somewhat agree. Concern responses were divided so that “Not Concerned” included “not concerned” and “minimally concerned” whereas “Concerned” included “moderately,” “very,” and “so concerned I do not want OpenNotes.” Data analysis was performed using SAS version 9.4.

## Results

A convenience sample of residents who were on their ambulatory block pre-implementation reached 37% (20/54) of resident physicians within our academic practice, with a 100% resident response rate. We obtained an overall response rate of 72% (31/44) for all our attending physicians. Of the combined group, 86% (44/51) agreed that they anticipated that OpenNotes could empower patients and help them better understand their respective care plans; 63% (44/51) expected that access to notes would make their patients worry more; 82% (42/51) were concerned that their patients would contact them with questions about the notes postimplementation. Both groups stated they anticipated changing their documentation about sensitive topics including cancer (31/51, 61%), mental health (36/51, 70%), and substance abuse (36/51, 70%).

**Figure 1:**
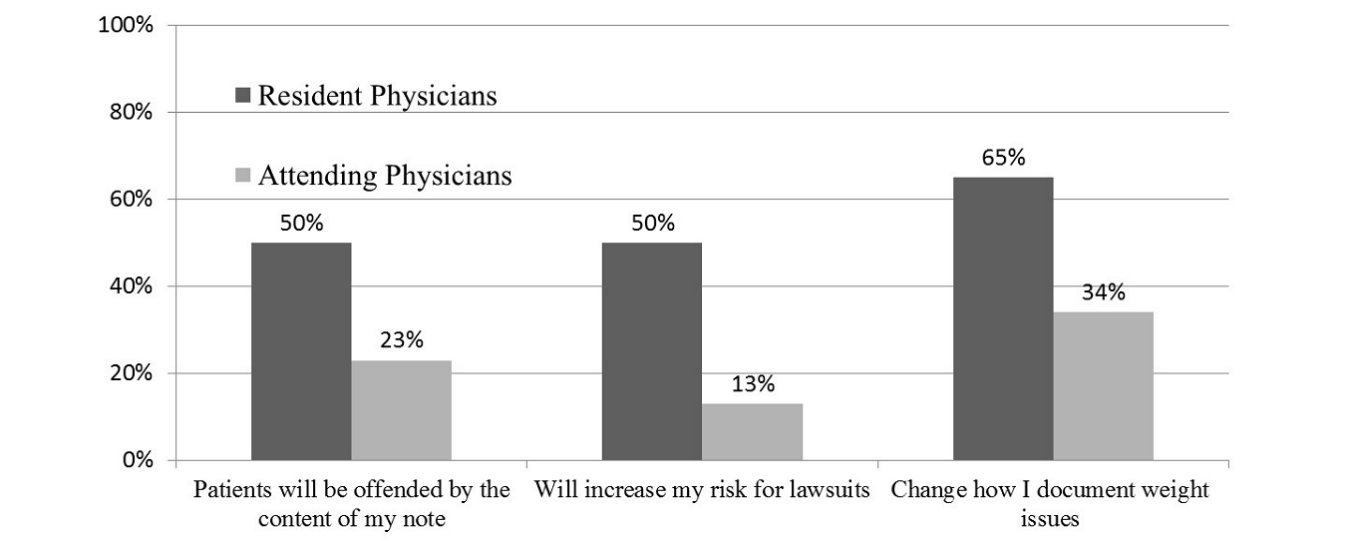
OpenNotes domains in which resident physicians report greater concerns than attending physicians. Results show those who either agree (agree and somewhat agree) or are concerned (moderately, very, and so concerned I do not want to open notes).

Between resident and attending physicians, there were some significant differences in survey responses. Resident physicians were more concerned than attending physicians about patients being offended by the contents of notes (50% [10/20] vs 23% [7/31]; *P*=.005). Resident physicians also perceived an increased risk of litigation (50% [10/20] vs 13% [4/31]; *P*=.01). Overall, 53% (16/31) of the attending physicians reported that they communicated almost daily with patients electronically compared with 0% (0/20) of the resident physicians (*P*<.0001). Regarding sensitive topics, the resident physicians felt more likely to change documentation about weight than the attending physicians (65% [13/20] vs 34% [10/30]; *P*=.03; Figure 1).

We analyzed a total of 30 separate entries for the 3 free response questions (questions 14, 31, 42). Two reviewers (DRN, MC) reviewed the comments and placed them within broad response categories. A major response category was concerns about more work with little yield or impact on patient outcomes but with an anticipation of increased patient empowerment. One provider stated that “notes will be longer, less helpful for reference later, as [I] may leave out things to make [the patient] happy.”

Another provider noted a personal experience stating, “Midwife let me look at my chart–allowed me to ask better questions.”

Another category increased patient confusion or concerns with note interpretation such as how to approach sensitive topics and medical terminology use. Comments included, “same issues as bedside rounds of mixing doctor speak and lay terms,” and “[I will be] less honest about feelings on sensitive topics.”

During the introduction session, resident physicians expressed significant concerns about how much additional work OpenNotes would create for them and how they would have to change major portions of their documentation. Many questions came up during the debriefing, primarily related to legal concerns and patient misinterpretation of their notes, which correlated with survey responses.

## Discussion

Although resident and attending physicians shared some of the same perceptions about OpenNotes, we found some significant differences. Both groups felt that OpenNotes had the potential to empower patients but were concerned about discussing sensitive topics in the notes. Our results corroborate with a recent qualitative analysis that showed that both attending and resident physicians were concerned about offending patients and potential litigation but felt that OpenNotes could be empowering [5]. However, our results showed that resident physicians expressed significantly more discomfort than attending physicians regarding litigation, discussing obesity, and offending their patients. Overall, our results are similar to those by Walker et al [1] that further validated our representative sample and revealed anticipation of improved patient communication and education, along with concerns about increased patient questions and litigation.

Differences in how OpenNotes was introduced to attending and resident physicians may have influenced the perception of OpenNotes. Our attending physicians were given several months to discuss potential opportunities and challenges surrounding the program, and their feedback was incorporated into logistical planning for the OpenNotes roll-out. Resident physicians, on the other hand, were introduced to OpenNotes as an initiative, regardless of their inputs. In the introduction session with the resident physicians, the overall response was of shock and concern about how much perceived additional work this would create for them and how they would have to significantly change their documentation style. To continue addressing these issues, we recommend open forums post-implementation to discuss the impact of OpenNotes on resident documentation or how patient feedback from the OpenNotes initiative could be helpful in alleviating many of these concerns. As seen in many post-implementation studies, most providers found that they made few, if any, changes to their notes post-implementation [4]. This also brings to light the need to include a more foundational curriculum addressing EHR and health portal use as patient engagement tools, medical–legal aspects of documentation, and setting of expectations for what changes, if any, this should make on their current documentation practices. It is possible that some of these differences are related to the fact that compared with the attending physicians, the resident physicians in this study communicated with their patients significantly less outside of the clinic. This could have led to more discomfort with granting patients electronic access to their own notes. Overall, as initiatives like OpenNotes become more common, it is important to find better ways to address concerns of the resident physicians surrounding patient access portals so that providers will use these portals as tools for patient empowerment. This study has several limitations: single-center study; small sample size; and consisted of only resident physicians.

In order to prepare trainees to be comfortable with EHR features such as patient access to provider notes, concerns about documentation, litigation, and increased electronic communication need to be addressed and additional curricula need to be developed, highlighting how to use these features to empower patients prior to implementation.

## Abbreviations

EHR: electronic health record
